# A Near‐Field Coupling Array Enables Parallel Imaging and SNR Gain in MRI

**DOI:** 10.1002/advs.202503481

**Published:** 2025-07-22

**Authors:** Zhiguang Mo, Shao Che, Feng Du, Enhua Xiao, Qiaoyan Chen, Nan Li, Sen Jia, Changjun Tie, Bing Wu, Xiaoliang Zhang, Hairong Zheng, Ye Li

**Affiliations:** ^1^ Paul C. Lauterbur Research Center for Biomedical Imaging Shenzhen Institutes of Advanced Technology Chinese Academy of Sciences Shenzhen 518055 China; ^2^ University of Chinese Academy of Sciences Beijing 101408 China; ^3^ Shenzhen Key Laboratory for MRI Shenzhen 518055 China; ^4^ Shanghai United Imaging Healthcare Co., Ltd Shanghai 201815 China; ^5^ Department of Biomedical Engineering State University of New York at Buffalo Buffalo New York 14203 USA

**Keywords:** magnetic resonance imaging, metasurfaces, near‐field coupling array, wearable devices, wireless coils

## Abstract

Wireless radiofrequency (RF) coils based on metasurfaces hold great promise for improving clinical magnetic resonance imaging (MRI) workflows by eliminating the need for cable connections to the patient bed and simplifying coil structures. A fundamental requirement for their clinical adoption is the ability to support parallel imaging, which reduces examination time and improves efficiency. Moreover, the implementation of parallel imaging often comes at the expense of image signal‐to‐noise ratio (SNR). Although several wireless RF coils incorporating metamaterials are reported, most do not support parallel imaging and many deliver suboptimal SNR performance. In this work, a novel wireless RF coil architecture is proposed, termed the near‐field coupling array (NFCA), which demonstrates both excellent SNR performance and robust parallel imaging capabilities. A general theoretical framework is presented that establishes a foundation for applying metasurface arrays in magnetic resonance RF coil design. The SNR expression for this architecture is derived from two perspectives, and its fundamental principles are validated through case studies. Experimental results show that the NFCA achieves a 66% SNR improvement over a conventional commercial RF coil architecture (i.e., a wired coil) while its average acceleration factor exceeds 94% of that of the commercial coil.

## Graphical abstract

This paper proposes a metasurface‐based radiofrequency (RF) coil architecture, providing a universal theoretical framework for the application of decoupled metasurface arrays in magnetic resonance imaging, with potential to address wireless connectivity issues in magnetic resonance imaging RF coils.

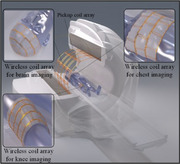

## 1 Introduction

**Figure 1:**
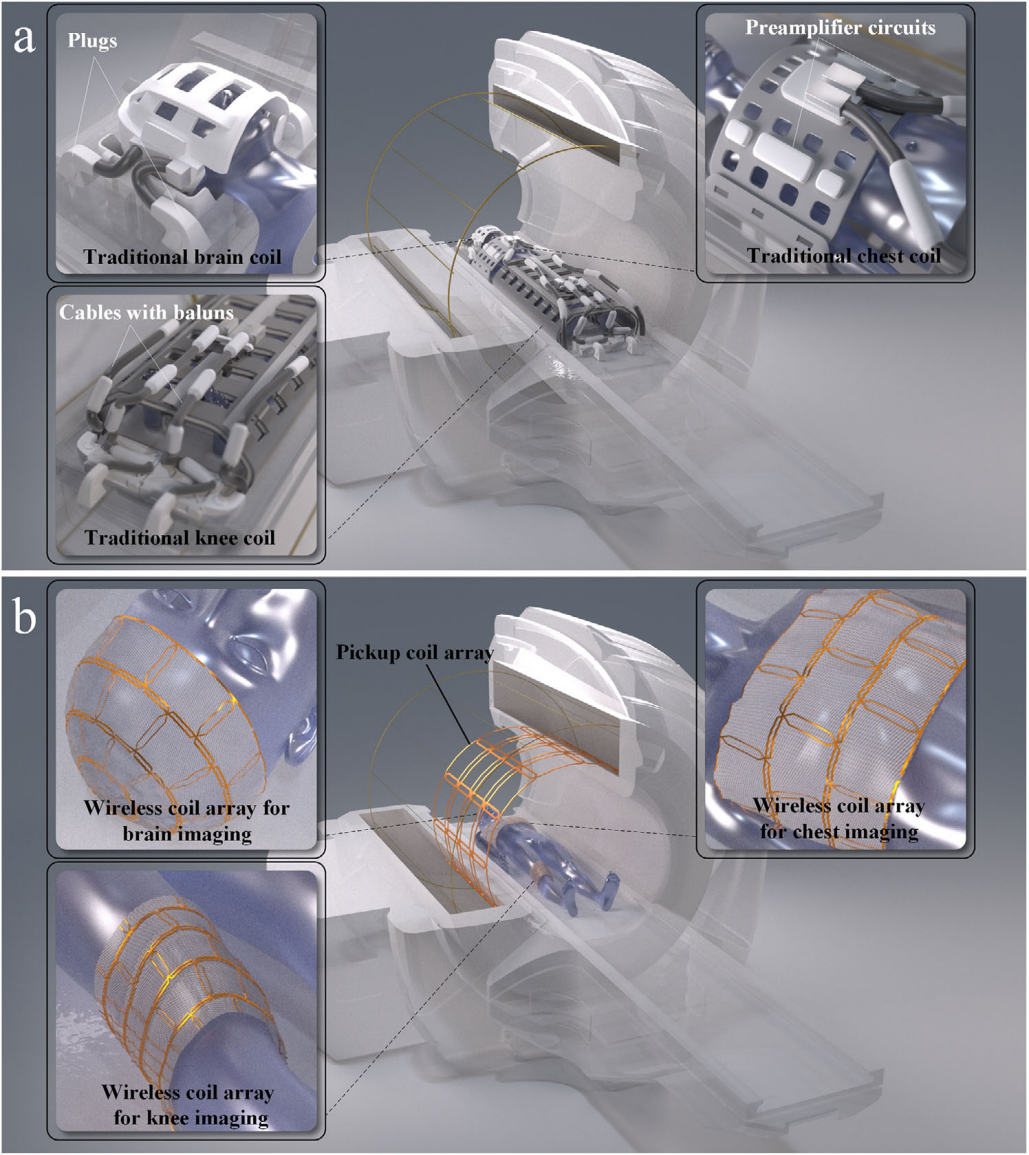
Comparison of magnetic resonance RF coil architectures. a) Traditional wired RF coils. This illustration depicts imaging coils utilized for the brain, knee, and chest regions, all characterized by bulky housings and cables connected to baluns and preamplifier circuits. b) The conceptual diagram of near‐field coupling array (NFCA) architecture proposed in this study. This system comprises a pickup coil array integrated within the magnet and a wireless coil array that is worn on various anatomical sites (such as the brain, knee, and chest, as illustrated). This design eliminates the necessity for cable connections between the wireless coils and the patient bed. Some electronic components are omitted in the figures.

Magnetic resonance imaging (MRI) is a nonionizing imaging modality that provides high‐resolution anatomical images of the human body through noninvasive scanning. The radiofrequency (RF) receive coil is a critical component in magnetic resonance systems responsible for signal acquisition, and its performance directly influences both imaging quality and speed. Over the past decades, the design of magnetic resonance RF coils has been primarily aimed at enhancing signal‐to‐noise ratio (SNR) performance and enabling parallel imaging capabilities.^[^ ^1^ ^]^ The enhanced parallel imaging capability allows for rapid image acquisition, while the elevated SNR mitigates the SNR degradation inherent to parallel imaging techniques.^[^ ^2^, ^3^, ^4^, ^5^, ^6^ ^]^ To achieve this, ultraflexible coil arrays^[^ ^7^, ^8^, ^9^ ^]^ and high‐density RF coil^[^ ^10^ ^]^ arrays have been developed. The former, while offering improved SNR due to its closer proximity to the imaging target, faces challenges related to its flexibility and examination convenience due to the presence of components, such as preamplifiers and cables. The latter, on the other hand, involves an increasing number of RF cables and connectors due to the higher number of channels.^[^ ^11^ ^]^ As these high‐density coil arrays approach their theoretical limits at a given field strength,^[^ ^12^ ^]^ the challenges related to the bulk and weight of the coils and cables become increasingly evident. These cables are designed to be long and thick to accommodate a variety of positioning needs and ensure reliability and low losses. To mitigate surface‐induced currents on the cables during system transmission, multiple bulky RF traps are added. When changing the scan area, the procedure involves disconnecting all connectors, replacing the coils—each weighing several kilograms, such as those used for the head or knee—and then reconnecting the connectors. Additionally, the cables present on the patient bed can pose a risk of burns to patients due to poor design, which may also contribute to their increased anxiety. The schematic of traditional RF coils is shown in **Figure** **1**a.

To address the challenges of wireless connectivity between RF coils and patient beds, several innovative approaches have been developed, each with distinct advantages and limitations. The first approach attempted to replace dedicated coils with a 128‐channel bore‐liner coil integrated within the magnet.^[^ ^13^ ^]^ While conceptually innovative, this “universal” remote body array proved impractical for routine imaging due to significant preamplifier noise coupling issues. A second strategy focused on integrating “on‐coil” communication antennas and associated electronic devices directly into the receive coil, replacing cables with communication methods, such as WiFi.^[^ ^14^, ^15^, ^16^ ^]^ Although this method successfully eliminated physical connections, it introduced new technical challenges, including the need for reliable wireless power supply for integrated electronics and precise synchronization of clock signals.^[^ ^17^ ^]^ Additionally, a third approach utilizing magnetic coupling‐based wireless technology has been developed to establish signal connections between the coil and the patient bed.^[^ ^18^ ^]^ While this method represents an advancement in replacing traditional plug connections, it fails to resolve the fundamental limitation of restricted coil positioning flexibility imposed by physical cable constraints.

An article published in Science in 2001 demonstrated that electromagnetic metamaterials with resonant properties can guide RF flux from an object to a remote receiver coil.^[^ ^19^ ^]^ This groundbreaking discovery laid the foundation for the application of metamaterials in the field of MRI RF coils. The use of metamaterials in MRI can be broadly categorized into two main scenarios. The first involves their use as auxiliary devices to enhance the SNR of traditional coils.^[^ ^20^, ^21^, ^22^, ^23^, ^24^, ^25^, ^26^, ^27^, ^28^ ^]^ In this configuration, the distance between the metamaterials and the conventional surface coils is typically maintained within the diameter of the coil. The second scenario focuses on leveraging metamaterials as a solution for implementing wireless RF coils.^[^ ^29^, ^30^, ^31^, ^32^, ^33^, ^34^ ^]^ In this setup, RF signals are amplified by resonant metamaterials and subsequently received by pickup coils, such as volume transmit coils (VTCs) or spine coils. Although the two configurations share similarities in their setup, they have fundamental differences in principle due to the impact of the pickup coil distance on the signal propagation path.

In previous studies, birdcage‐shaped metamaterials have been reported to perform effectively for imaging at low field strengths and small anatomical regions, such as the wrist.^[^ ^30^ ^]^ However, under 3T field strength or in large field‐of‐view conditions, the SNR of such globally resonant metamaterials is typically lower than that of conventional coils.^[^ ^29^, ^33^, ^35^, ^36^ ^]^ In addition, previous solutions typically employed VTCs or spine coils as pickup coils. Due to limitations in channel count or suboptimal SNR performance, these systems face constraints in parallel imaging capabilities.^[^ ^32^ ^]^ The insufficient SNR and parallel imaging capabilities constitute the primary obstacles hindering the clinical application of metamaterials. At this stage, there is an urgent need for a theoretical framework to guide the design of metamaterials, in order to demonstrate the feasibility of their practical application in the field of clinical magnetic resonance RF coils.

In this paper, we present the architecture of a NFCA, which comprises two primary components: 1) a decoupled metasurface structure, referred to as the wireless coil array, that is worn on the patient's body; and 2) a wired pickup coil array, designed as a general‐purpose module that can be implemented as an upgradeable component integrated into the patient bed, and may potentially be factory‐integrated into the scanner as a bore liner in the future. The conceptual diagram of the NFCA employing a bore‐liner‐type pickup coil array is shown in Figure 1b. Unlike other solutions, the pickup coil used in NFCA is a multichannel array that surrounds the imaging target, while the decoupled metasurface provides spatially distinct signals. This combination is key to enabling NFCA to achieve exceptional parallel imaging performance.

To validate our concept, we derived the SNR expression for the NFCA and confirmed its accuracy in phantom study. Subsequently, we developed an NFCA and compared its SNR and parallel imaging capabilities with those of its pickup coil array alone and a commercially available clinical knee coil through in vivo studies. The results suggest that the proposed NFCA has the potential to meet the clinical needs for SNR and parallel imaging capabilities, making it a promising new solution for clinical applications. A part of this paper has been reprinted on arXiv.^[^ ^37^ ^]^

## 2 Theoretical Analysis of SNR in NFCA

In this section, we first derived the SNR expression for NFCA based on the principle of reciprocity. We then applied Kirchhoff's laws to derive the formula from an alternative perspective. Through the theoretical derivation and validation of these two methods, we aim to deepen our understanding of the signal combination mechanisms in NFCA and the spatial signal anisotropy of metasurfaces. The conclusions were validated through simulations and measurements using a phantom. Additionally, we developed a prototype of the NFCA and conducted an in vivo study to investigate its potential as a viable alternative to existing commercial RF coils.

**Figure 2:**
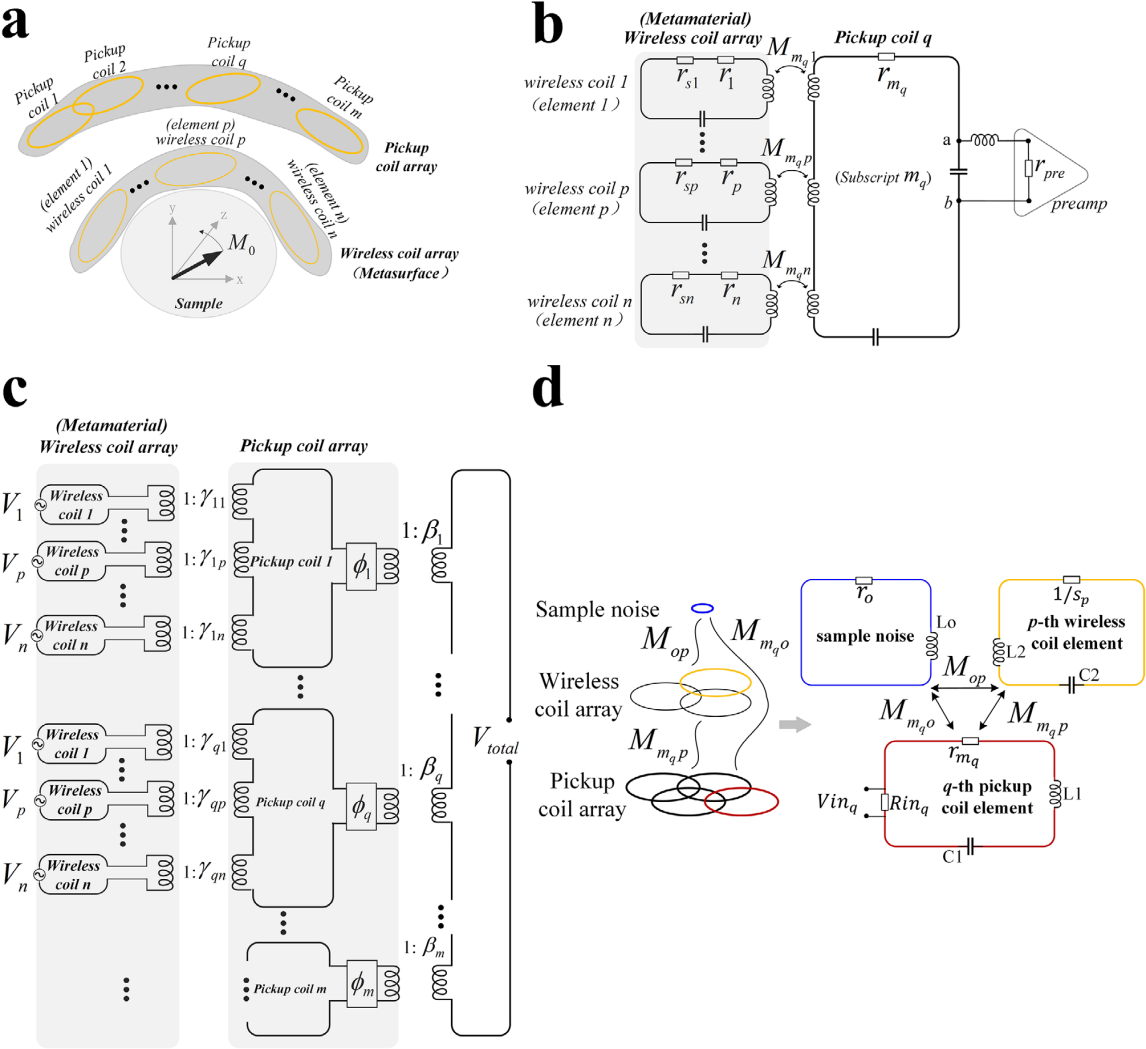
Schematic diagrams of the NFCA. a) The pickup coil array consists of m elements, and the wireless coil array (metasurface) consists of n elements. *M*_0_ is the magnitude of the magnetization density rotating at the Larmor frequency *ω*. The sample represents the object to be imaged. b) The circuit schematic of the *q*th pickup coil and the wireless coil array. The wireless coil array mentioned in the paper refers to the decoupled metasurface array, where the *p*th coil represents the *p*th element of the decoupled metasurface. (To avoid distracting the reader, under the assumption that both the wireless coils and pickup coils are in series resonance, the values of the capacitors and inductors that do not affect the calculation results are not shown in the diagram.) c) Schematic diagram of signal transmission and combination in NFCA. d) Schematic and circuit diagram of the relationship between the pickup coil elements, wireless coil elements, and the equivalent noise loop.

Before conducting the theoretical analysis, we need to provide a detailed definition of the NFCA. The proposed NFCA is an RF coil architecture designed to operate during the signal reception phase. It consists of two components: a pickup coil array and a decoupled metasurface, known as the wireless coil array, as shown in **Figure** **2**a.

An ideal NFCA should meet the following conditions:

1. The wireless coil needs to be positioned close to the subject to improve the SNR.
2. The direct coupling between the elements of the wireless coil array (metasurface) can be neglected. Which means the alternating current signal resonates independently in each element.
3. The wireless coil needs to include a detuning module to minimize its impact on the transmit field B1+ and the specific absorption rate (SAR).
4. The direct coupling between the elements of the pickup coil array can be neglected.
5. The pickup coils are typically placed farther away from the imaging object than their own diameter, while the wireless coil array is always positioned close to the imaging object. Given this spatial arrangement, the direct signal received by the pickup coil from the sample can be considered negligible.

Point 1 should be followed because the signal rapidly attenuates as the distance from the conductor increases. However, a space larger than the conductor's diameter is usually required to prevent the formation of a capacitive effect between the conductor and the load.

For point 2, one or more of the following methods—overlapping decoupling,^[^ ^38^ ^]^ induced current elimination (ICE) decoupling,^[^ ^39^ ^]^ and other special designs^[^ ^32^ ^]^ to reduce surface currents in the loops—can theoretically achieve this goal.

For point 3, an adaptive parallel resonant circuit module is a feasible implementation approach, as demonstrated in the Experimental Section of this paper.

For point 4, The decoupling between the pickup coils is typically achieved through a combination of overlapping decoupling and the use of a low‐resistance preamplifier. Figure 2b illustrates the circuit diagram between the *q*th pickup coil and the n‐element wireless coil array.

In this diagram, *r_p_* ​ represents the equivalent resistance of the coil noise, while *r_sp_* corresponds to the equivalent resistance of the sample noise for the *p*th wireless coil. $M_{m_{q}p}$ is the mutual inductance coefficient between the *p*th wireless coil and the *q*th pickup coil, $r_{m_{q}}$ is the circuit resistance of the *q*th pickup coil and *r*_pre_ refers to the input resistance of the preamplifier of the pickup coils. When *r*_pre_ is low, a parallel resonant circuit will be formed between points a and b. It is equivalent to a large resistor, which prevents current from flowing through the pickup coil (A typical value for a preamplifier input impedance of 5 ohms, at which the equivalent resistance between points a and b is ≈1–2 kΩ^[^ ^38^ ^]^). As the current decreases, coupling suppression between different pickup coils will be achieved. Theoretically, this effect will increase as *r*_pre_ decreases.

The setup for point 5 is intended to align with the expected usage scenario of NFCA, where the pickup coil is integrated within the magnet or any other positions that are relatively far from the sample, while the wireless coil array is worn by the subject. When the distance between the pickup coil and the sample is greater than the diameter of the pickup coil, the signal transmission will follow the path from the sample to the wireless coil and then to the pickup coil, without being directly received by the pickup coil.

### 2.1 SNR Derivation Using Reciprocity Theory

To provide an intuitive representation of how the signals induced by the n wireless coils are transmitted to the pickup coil array and ultimately combined, we refer to the approach outlined in ref. [38] In this method, the amplification and attenuation of the signals during transmission over the RF link are modeled using an ideal transformer, as illustrated in Figure 2c. The total signal voltage can be obtained from the signals on all the pickup coils through lossless phase shifters and transformers

(1) $$V_{\mathrm{total}}^{\mathrm{pickup}}=\omega^{2}M_{0}V\left|\sum_{q=1}^{m}\sum_{p=1}^{n}\frac{M_{m_{q}p}}{r_{p}+r_{sp}}\vec{B}1_{wp}e^{j\theta_{p}}\right|\cos\left(\omega t+\psi -\frac{\pi }{2}\right)$$

where *ω* is the larmor frequency, *M*_0_ is the magnetization density, *V* is the volume of the voxel, ${\vec{B1}}_{wp}$ is the spatially distributed transverse magnetic field generated by unit current in the *p*th wireless coil, θ*_p_* is the angle of the RF magnetic field measured from some fixed reference in the laboratory frame, and ψ is the arbitrary phase of the rotating nuclei. In this paper, terms multiplied by $e^{j\theta_{p}}$ are assumed to take only the real part by default.

The cross‐coupling noise caused by the electric field between different channels in the sample is mainly received by the wireless coil array, rather than directly by the pickup coils. Therefore, the total input impedance is expressed as

(2) $$Z\;in_{\mathrm{total}}=\sum_{q=1}^{m}\sum_{p=1}^{n}\frac{\lambda_{\mathrm{total}}{\left(\omega M_{m_{q}p}\right)}^{2}}{r_{p}+r_{sp}}$$

*λ*_total_ is a correction factor applied to the input impedance, accounting for the impact of the equivalent resistance of the pickup coil on the overall input impedance. The range of *λ*_total_ is from 1 to infinity. When the pickup coil is positioned in close proximity to the wireless coil, the strong mutual inductance allows the coil noise of the pickup coils to have a negligible effect on the input impedance. In this case, *λ*_total_ is equal to 1. When the pickup coil is placed farther away from the wireless coil, the equivalent impedance contributed by the wireless coil decreases, and the coil noise of the pickup coil cannot be neglected. In this case, *λ*_total_ is greater than 1. For a 70 cm bore MR scanner, a value of 1.5 is typical for a 12‐channel pickup coil array with a side length of 20 cm. A typical value is 1.5 for a 12‐channel pickup coil array used as a bore‐liner in a 70 cm bore MR scanner.

As the SNR at the output is defined as the ratio of the instantaneous signal to the root mean square noise voltage^[^ ^38^ ^]^

(3) $$SNR=\frac{\left|V_{\mathrm{out}}\right|}{\sqrt{4kT\Delta fRe(Zin)}}$$

The SNR expression can be calculated by substituting Equations (1) and (2) into Equation (3)

(4) $$SNR=\frac{\omega M_{0}V\left|\vec{B}1_{\mathrm{total}}\right|}{\sqrt{4kT\Delta f\cdot \lambda_{\mathrm{total}}\left(\sum_{p=1}^{n}r_{p}+\sum_{p=1}^{n}r_{sp}\right)}}$$

In comparison with the expression for conventional wired coils, the primary distinctions are the terms *λ*_total_ and ${\vec{B1}}_{total}$. Specificanlly, *λ*_total_ contributes to a decrease in SNR as a result of the separation between the pickup coil and the wireless coil. To mitigate this effect, employing low‐loss pickup coils may offer potential improvements in SNR. Here, ${\vec{B1}}_{total}$​ represents the total B1‐ field^[^ ^40^ ^]^ of the NFCA, which is determined by the combination of the individual B1‐ fields of each wireless coil (metasurface element). For simplicity and readability, B1 is used to denote B1^−^ throughout this paper, as B1⁺ is not involved in the SNR‐related theoretical analysis discussed in this paper

(5) $$\vec{B}1_{\mathrm{total}}=k_{t1}\vec{B}1_{w1}e^{j\theta_{1}}+\cdot \cdot \cdot +k_{tp}\vec{B}1_{wp}e^{j\theta_{p}}+\cdot \cdot \cdot k_{tn}\vec{B}1_{wn}e^{j\theta_{n}}$$

where

(6) $$k_{tp}=\frac{\sum_{q=1}^{m}M_{m_{q}p}}{\sqrt{\sum_{q=1}^{m}\sum_{p=1}^{n}M_{m_{q}p}^{2}}}$$

We can find from the expressions that the signal distribution of the NFCA is the result of the combination of the B1 fields from different wireless coils, weighted by coefficients. These weights depend on the mutual inductance coefficients between each wireless coil (or each unit of the decoupling metasurface) and all pickup coils.

Assuming that the signal transmission path between each wireless coil element and each pickup coil element is represented by *k_qp_*, where

(7) $$k_{qp}=\frac{M_{m_{q}p}}{\sqrt{\sum_{q=1}^{m}\sum_{p=1}^{n}M_{m_{q}p}^{2}}}$$

The sum of the squares of all *k_qp_* equals 1. This indicates that the coefficients of the B1 field—determined by the various coupling paths and wireless coils—are automatically normalized prior to superposition

### 2.2 SNR Derivation Using Kirchhoff's Laws

Reference ^[^ ^41^ ^]^ presented SNR expressions based on Kirchhoff's laws for a pickup coil element in combination with one or two mutually coupled wireless coils, showing that the results were consistent in form with traditional formulations. This inspired us to analyze the case of n wireless coil elements combined with m pickup coil channels based on Kirchhoff's laws.

In this paper, we adopted the damping model shown in ref. [42] to simulate the sample losses caused by the imaging object. The sample noise is modeled as a resonant wire loop coupled to the coil, with an resistance *r*_o_​ on the loop. Consequently, the sample loss of the *p*th wireless coil can be represented as

(8) $$r_{sp}=\frac{\omega^{2}M_{op}^{2}}{r_{o}}$$

where *M_op_* is the mutual inductance coefficient between the *p*th coil and the equivalent current loop of the sample noise. This value is proportional to *ω* ^2^, consistent with the frequency dependence of induced RF losses in the human body.^[^ ^43^ ^]^

When considering the interaction between the m‐channel pickup coil array and the n‐element wireless coil array, their schematic and circuit diagram with sample noise can be represented as shown in Figure 2d. Sample noise is denoted by the subscript “*o*,” with “*p*” representing the *p*th wireless coil element and “*m_q_*” referring to the *q*th pickup coil element. *M_op_*, $M_{m_{q}o}$, and $M_{m_{q}p}$ denote the mutual inductance coefficients between each pair of these elements, respectively. The relationship can also be expressed in matrix form as follows

(9)

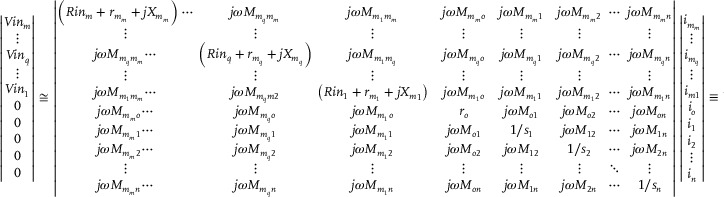

where *Vin_q_* is the electromotive force (EMF) applied to the *q*th pickup coil element, *Rin_q_* is the equivalent source impedance and $r_{m_{q}}+jX_{m_{q}}$ is the impedance caused by the *q*th pickup coil conductor and components on the coil. Clearly, the three subscripts of the mutual inductance coefficient M−*m_q_*, *o*, and *p* (where *p* is a natural number between 1 and *n*)—represent the *q*th pickup coil, the equivalent current loop of the sample noise, and the *p*th wireless coil element, respectively. The subscripts of the current *i* have the same meaning. We use the resistance *r_o_* ​ to represent the resistance of the equivalent current loop for sample noise, and the conductance 1/s*_p_* to represent the resistance of the *p*th wireless coil element, which will simplify our calculations.

To obtain the SNR of the NFCA, we need to calculate the total input impedance Zin and the total induced voltage ξ of the pickup coil based on their relationship, as defined in Equation (3). As defined earlier for the NFCA, well‐designed wireless coils are considered to be well decoupled. Accordingly, the *M* values in the matrix representing the mutual inductance coefficients between pickup elements and between wireless coil elements are all set to zero. The total EMF signal can be expressed as

(10) $$\begin{array}{ccc} & & \;\;\xi_{\mathrm{total}}\\ & & \;\;=\frac{\sum_{p=1}^{n}\omega^{2}M_{m_{1}p}M_{xp}s_{p}r_{o}+\cdot \cdot \cdot +\sum_{p=1}^{n}\omega^{2}M_{m_{q}p}M_{xp}s_{p}r_{o}+\cdot \cdot \cdot +\sum_{p=1}^{n}\omega^{2}M_{m_{m}p}M_{xp}s_{p}r_{o}}{r_{o}+\sum_{p=1}^{n}\omega^{2}M_{op}^{2}s_{p}}\cdot i_{x} \end{array}$$

While the total input impedance is

(11) $$\begin{array}{c} Zin_{\mathrm{total}}=\sum_{p=1}^{n}\frac{\omega^{2}r_{o}\left(\sum_{p=1}^{n}M_{m_{1}p}^{2}s_{p}+\cdot \cdot \cdot +\sum_{p=1}^{n}M_{m_{q}p}^{2}s_{p}+\cdot \cdot \cdot +\sum_{p=1}^{n}M_{m_{m}p}^{2}s_{p}\right)}{\lambda_{\mathrm{total}}\left(r_{o}+\sum_{p=1}^{n}\omega^{2}M_{op}^{2}s_{p}\right)}\;\;\;\; \end{array}$$

According to the definition of mutual inductance, $M_{x_{p}}$ can represent the magnetic flux in the ideal current loop generated by the *p*th wireless coil carrying a unit current, which can be expressed as $\delta A\cdot \vec{B}1_{w_{p}}e^{j\theta_{p}}$, when the wireless coil and the ideal current loop share the same normal direction, θ_*p*_ is 0. Substituting $i_{x}=\frac{M_{0}\delta V}{\delta A}\;$ into Equation (10), and since the differences in coil noise and sample noise observed by different wireless coils are much smaller than the variations in the mutual inductance coefficient, we extract the terms related to r*_p_* and r*_sp_* as a common factor for simplification. Thus, the SNR of the NFCA by substituting Equations (10) and (11) into Equation (3)

(12) $$SNR=\frac{\omega M_{0}\delta V\left|\vec{B}1_{\mathrm{total}}\right|}{\sqrt{4kT\Delta f\cdot \lambda_{\mathrm{total}}\left(\sum_{p=1}^{n}r_{p}+\sum_{p=1}^{n}r_{sp}\right)}}$$

Which is the same as the result obtained using the principle of reciprocity. The meanings of the symbols herein are consistent with those in Equation (4). And a symbol reference chart can be found in the Supporting Information.

## 3 Result

### 3.1 Bench Testing and SAR Measurement

**Figure 3:**
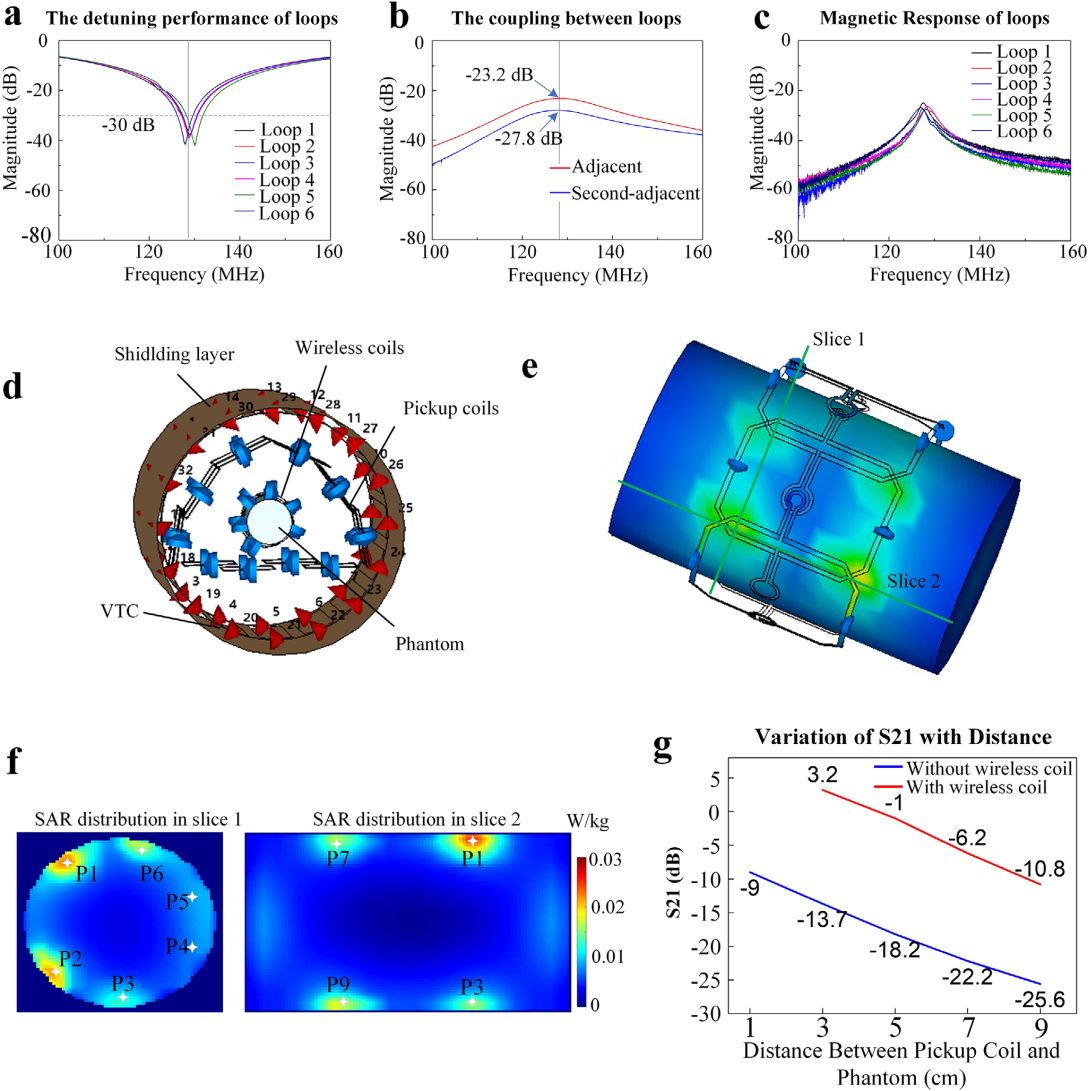
Bench testing and SAR simulation results. a) The detuning performance of the wireless coils. b) Coupling between adjacent and next‐adjacent wireless coils. c) Magnetic response of individual elements in the wireless coil array. d) Schematic diagram of the SAR simulation setup. e) 3D results of the SAR simulation. f) SAR distribution along slice 1 and slice 2. The input power for the simulation was set to 1 W. The white star‐marked points in the figure indicate the locations where fiber optic probes were placed during the heating test. g) Transmission coefficient S21 as a function of distance with and without the wireless coil.

At the operating frequency, the transmission coefficient (S21) of each unit in the wireless coil array was below −30 dB (**Figure** **3**a), indicating that each unit maintained a high‐impedance state when the diode was forward‐biased. This reduces direct energy coupling from the transmit coil. The S21 between adjacent units was below −23 dB, and that between next‐adjacent units was below −27 dB, suggesting low coupling between neighboring elements during the receive phase. This allows the metasurface units to resonate more independently (Figure 3b). The magnetic response obtained using a dual‐loop probe further confirmed that each unit could resonate relatively independently (Figure 3c).

The 3D electromagnetic simulation results of the phantom show that, under the setup illustrated in Figure 3d, regions of elevated local SAR are primarily located within the phantom, beneath the overlapping areas of the wireless coil conductors (Figure 3e). To verify whether the SAR in these regions exceeds safety limits, 12 fiber optic probes were placed at the corresponding locations (some of which are shown in Figure 3f) to measure the temperature rise. More detailed data can be found in the Supporting Information. After scanning with a high‐duty‐cycle sequence for 30 min, the maximum observed temperature increase was 1.1 °C, and the corresponding SAR value is ≈2.5 W kg^−1^, which remains well below the 10 W kg^−1^ safety threshold.^[^ ^44^ ^]^

Figure 3g illustrates the signal amplification effect of the wireless coil array during signal transmission. The experimental results indicate that at distances of 3, 5, 7, and 9 cm from the phantom, the signal strength received by the pickup coil with the presence of the wireless coil is 15–17 dB higher than that without the wireless coil.

### 3.2 Simulation and Experimental Validation Using a Phantom

**Figure 4:**
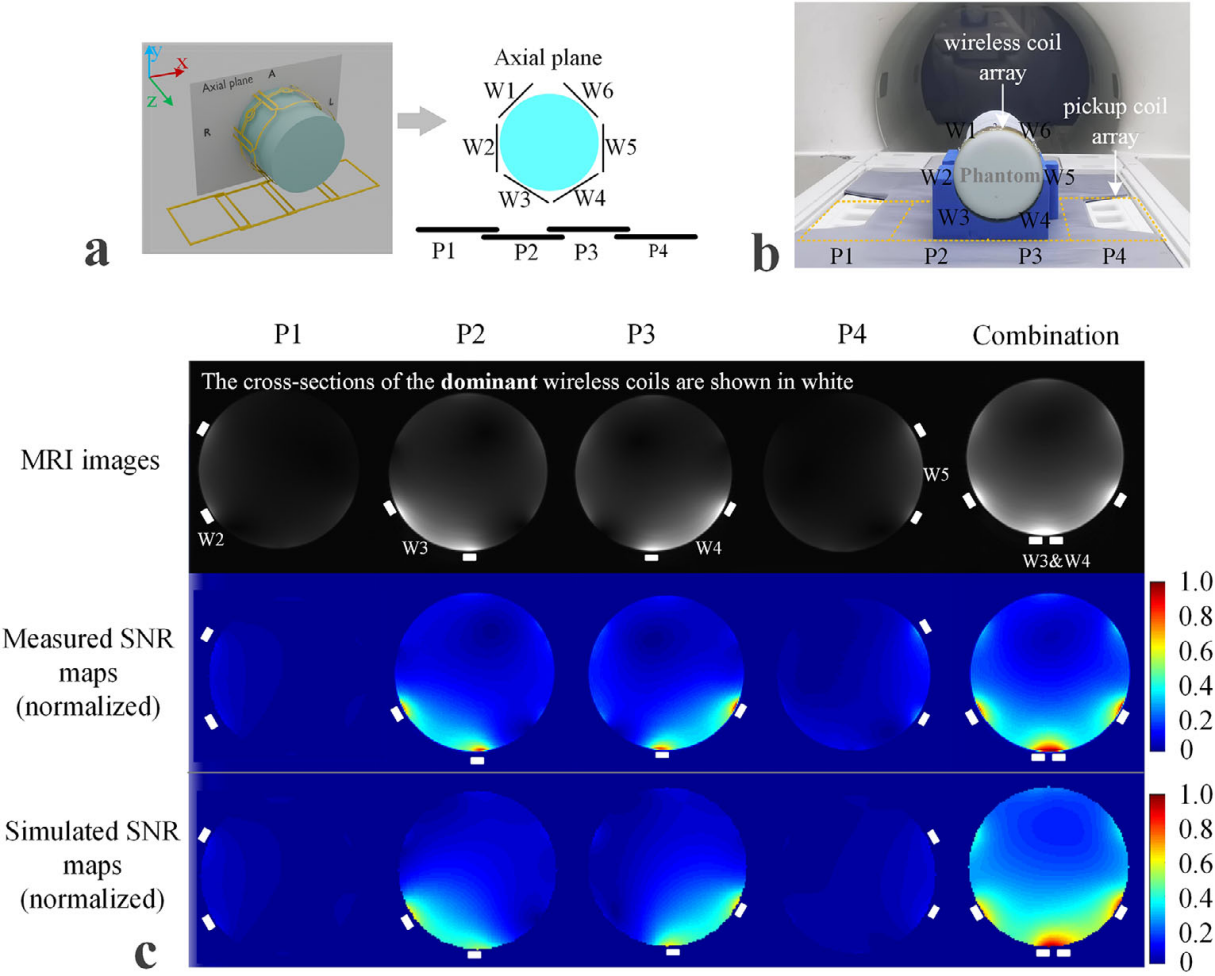
Simulation and experimental validation of signal combination using a phantom. a) Schematic diagram of the simulation setup. b) Schematic diagram of the MRI experiment. c) MRI images, measured SNR maps, and simulated SNR maps obtained from individual receptions using each pickup coil channel, as well as from collective receptions using all pickup coil channels. W3 and W4, which have a larger mutual inductance with the pickup coil, make significant contributions to the signal, while other wireless coils with smaller mutual inductance contribute less, which is consistent with **Table** **1**. All SNR maps have been normalized to their maximum values.

Theoretical analysis indicates that the overall B1 field distribution of the NFCA results from a weighted combination of the B1 fields of individual wireless coils, with weights determined by their mutual coupling coefficients. Similarly, the SNR distribution is expected to follow this pattern. To validate this, we designed a dedicated experiment. We employed a combination of four pickup coils and a wireless coil array (metasurface), among which the mutual inductance coefficients varied significantly, making it more convenient to validate the conclusions. The configurations for the simulation and practical testing are shown in **Figure** **4**a,b, respectively.

As shown in Table 1, when using different individual pickup channels as receivers, the coil elements in the wireless coil array with the highest mutual inductance coefficients are W2, W3, W4, and W5, respectively. When all pickup channels are used as receivers, the two wireless coil elements with the largest mutual inductance coefficients are W3 and W4. The B1 fields of these wireless coils (or metasurface elements) dominate the overall B1 field, as illustrated in Figure 4c. The dominant wireless coils are highlighted in white in the figures. Likewise, when all pickup coil channels are utilized collectively as receivers, the overall SNR map is also dominated by W3 and W4, which have the highest mutual coupling coefficients with the entire pickup coil array. This phenomenon is consistently observed in the MRI images, measured SNR maps, and simulated SNR maps.

**Table 1:** Mutual inductance between pickup coils and elements of wireless coil.

	W1	W2	W3	W4	W5	W6	Dominant elements
P1^*^	1.1^*^	5.7	1.1	1.3	0.9	1.1	W2^*^
P2	4.3	4.9	36	1.8	5.1	4.1	W3
P3	4.4	6.6	2.9	36	3.1	3.8	W4
P4	0.9	1.3	2.2	1.1	6	0.7	W5
Combination^*^	10.7	18.5	42.2	40.2	15.1	9.7	W3 & W4

^*^ P1 ‐ P4: Pickup coil elements.

^*^ W1 ‐ W6: Wireless coil elements.

^*^ Combination: All pickup coil elements were used.

^*^ All values in the table are in nanohenries (nH).

**Table** **2** presents the measured and simulated mean SNR and coefficient of variation (COV) within slices and volumes, where the volume consists of 20 slices. The results indicate good agreement between simulation and measurement. This experiment demonstrates that the B1 field, which represents the SNR distribution of the wireless coils (metasurfaces) in the NFCA, is formed by a weighted combination of components, as described in Equations (4) and (12).

**Table 2:** Simulated and measured normalized SNR and COV in phantom imaging.

Metric	Measured values	Simulation values
Slice mean SNR (Normalized)	0.237	0.264
Slice COV^a)^ [%]	62	57
Volume mean SNR (Normalized)	0.239	0.265
Volume COV [%]	61	57

^a)^ COV: Coefficient of variation.

### 3.3 In Vivo Experiment Validation

#### 3.3.1 SNR and Noise Correlation

**Figure 5:**
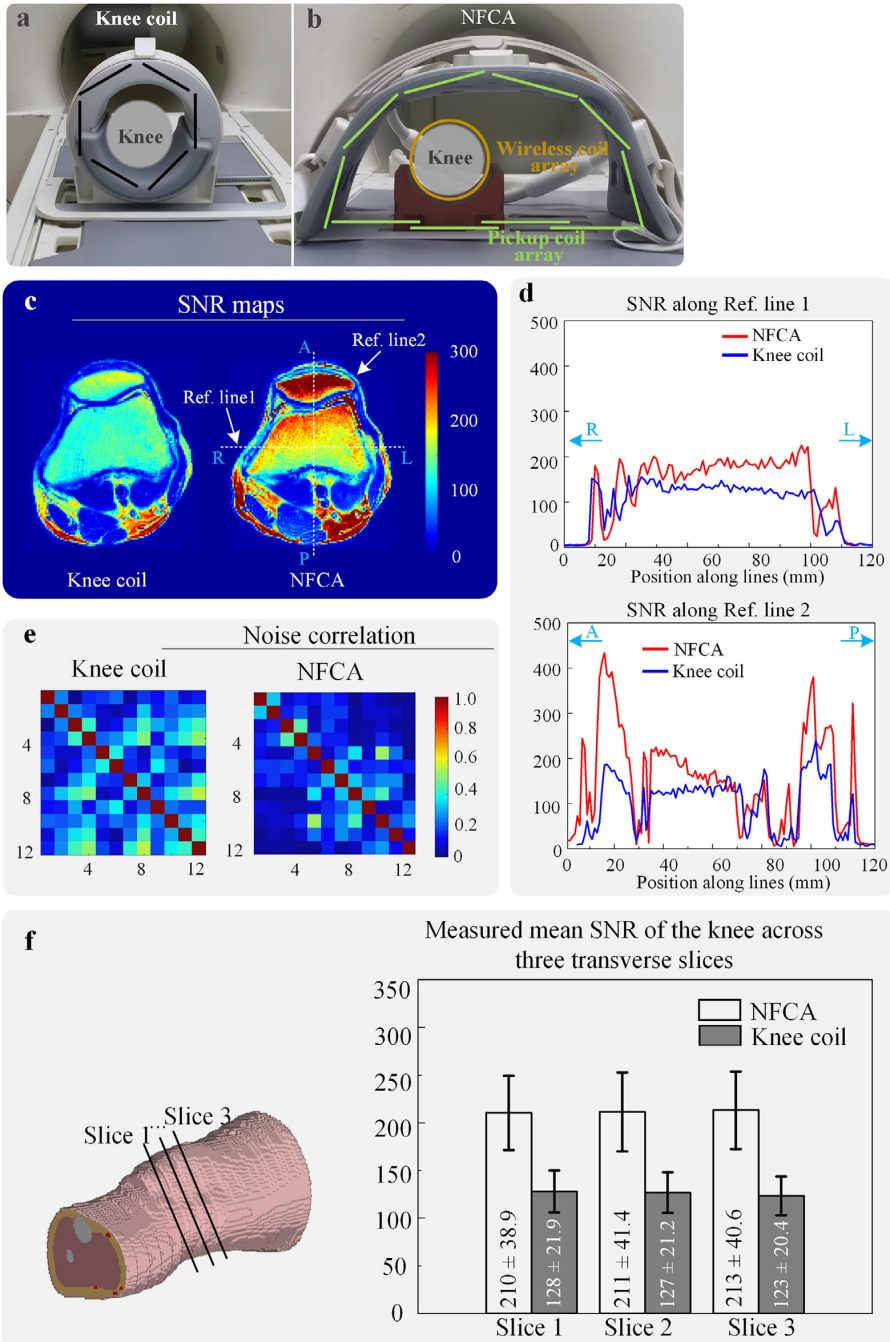
SNR Imaging Experiments. a) Schematic diagram of the experimental setup for the knee coil. b) Schematic diagram of the experimental setup for the NFCA. c) SNR maps of the cross‐sectional knee MRI images obtained using these setups. d) Comparison of SNR values along two reference lines (Ref. line 1 and Ref. line 2). e) Noise correlation matrices obtained using the two types of receivers. f) Comparison of SNR between NFCA and the knee coil. Data are presented as mean ± SD (*n* = 9 volunteers).

In the in vivo experiment, we compared the SNR obtained using the NFCA and a conventional knee coil (a 12‐channel surface coil array), as illustrated in **Figure** **5**a,b. To avoid the confounding effect of differing channel counts in the SNR comparisons, we activated only 12 channels of the pickup coil array in this section, consistent with the number used in the knee coil. The NFCA exhibits a significant SNR advantage over the knee coil, particularly in regions close to the surface (Figure 5c). The SNR values along the two reference lines also indicate this trend (Figure 5d). And the noise correlation values obtained with the NFCA and knee coil indicate that the addition of the wireless coil array did not cause any significant deterioration in the noise correlation coefficients of the pickup coil array (Figure 5e). In Figure 5f, nine volunteers with heights ranging from 153 to 181 cm were recruited for repeated experiments. Despite individual variability, the NFCA consistently demonstrated an average SNR improvement of ≈66% (*p* < 0.001).

**Table** **3** presents the average signal‐to‐noise ratio (SNR) and coefficient of variation (COV) for a single imaging slice and the imaging volume from a single volunteer in the in vivo study. The selected slice corresponds to the one shown in Figure 5c. The imaging volume consists of 20 slices, covering ≈8 cm along the knee direction. These data indicate that, for some individuals, the NFCA can achieve up to a 71% improvement in SNR compared to the knee coil, while the difference in COV remains within 5%.

**Table 3:** Mean SNR values and COV obtained from a single volunteer.

Metric	Knee coil	NFCA
Slice mean SNR	94.8	162.5
Slice COV^a)^ [%]	65.4	68.0
Volume mean SNR	94.4	161.3
Volume COV [%]	66.5	66.4

^a)^ COV: Coefficient of variation.

#### 3.3.2 G‐Factor and Anatomical Images

**Figure 6:**
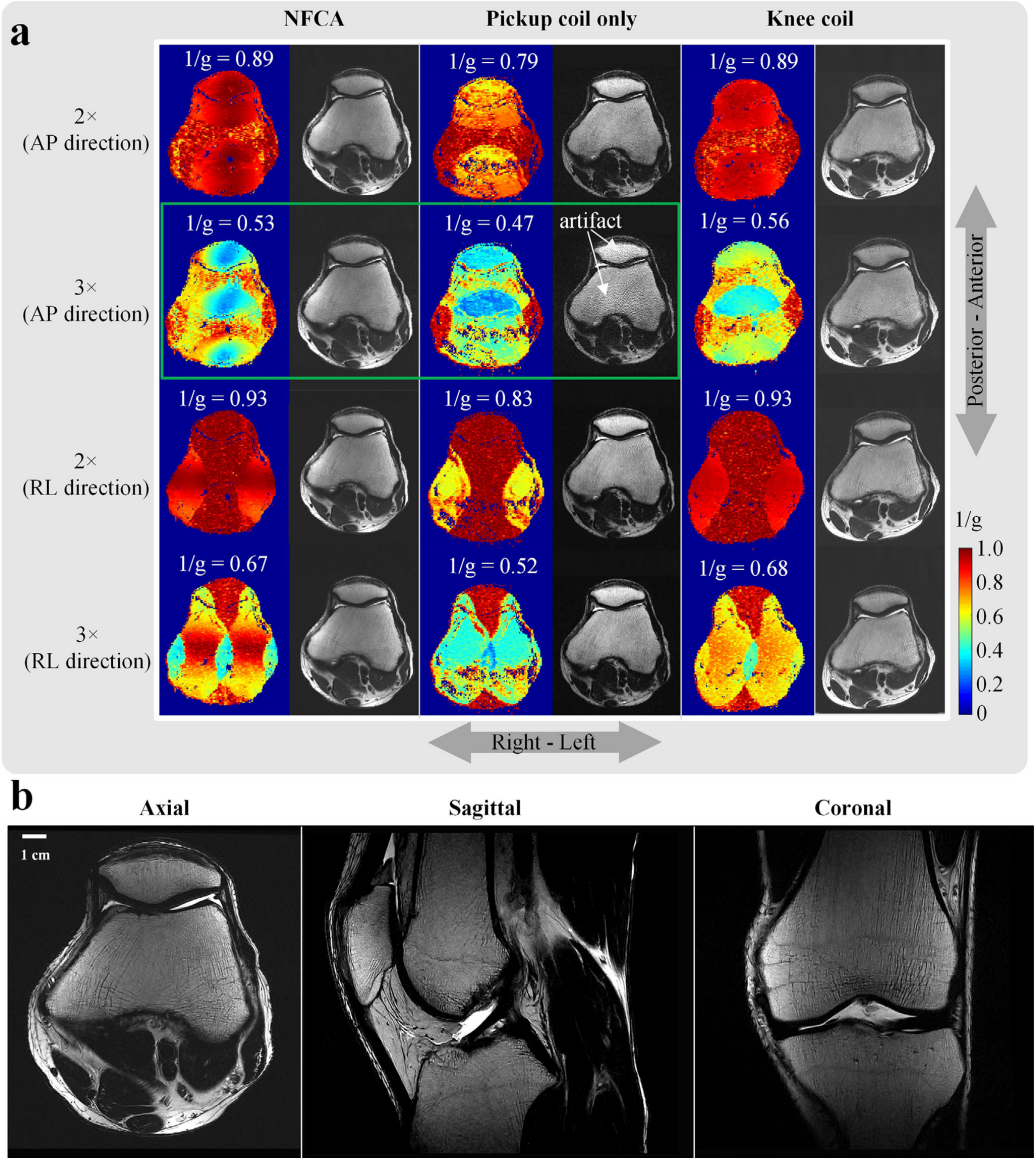
In vivo study. a) Comparison of the parallel imaging capabilities of NFCA, its pickup coil, and the knee coil. 1/g maps under identical acceleration conditions are shown (g represents the mean g‐factor, and higher 1/g values indicate better parallel imaging performance). Next to the 1/g maps are the anatomical images obtained from the MR system's built‐in software. b) The T2‐weighted MRI images of the cross‐sectional, sagittal, and coronal planes obtained using NFCA have a high resolution of 0.33 mm × 0.33 mm × 2 mm.

To validate the parallel imaging capability of the NFCA, axial imaging of a volunteer's knee was performed using the NFCA, a knee coil, and its pickup coils only, as shown in **Figure** **6**a. Parallel imaging was conducted with acceleration factors of 2 and 3 in the AP and RL directions, respectively. The colored images, which represent the 1/g maps, provide an intuitive visualization of the data.

The results indicate that the parallel imaging capability of the NFCA, as measured by the 1/g‐factor (highlighted in white), has improved by 12%–22% compared to the pickup coil only, achieving parallel imaging performance equivalent to the knee coil (2× acceleration) or exceeding 94% of its performance (3× acceleration). This demonstrates that the parallel imaging capability of the NFCA has the potential to match that of traditional coils. In the MRI results with a threefold acceleration in the AP direction, spotty artifacts caused by the insufficient acceleration performance of the pickup coil only can be observed (highlighted by green boxes). This suggests that the wireless coil array plays an important role in achieving the high parallel imaging performance of the NFCA. On the other hand, in the AP direction, the g‐factor of the NFCA is slightly inferior to that of the knee coil, which may be attributed to the lower density of coil elements in the AP direction for the pickup coil.

The SNR performance allows the NFCA to achieve imaging at a resolution of up to 0.33 mm × 0.33 mm × 2 mm. Figure 6b illustrates the MR images of three typical cross‐sections of the knee.

## 4 Discussion

In this study, we have introduced the concept of the NFCA to provide a reference paradigm for the next generation of metamaterial‐supported wireless RF coils. Unlike traditional coils connected to the patient bed via cables, the NFCA facilitates signal acquisition through a combination of a patient‐worn wireless coil (metasurface) and a pickup coil array integrated into the magnet. The NFCA eliminates the need for frequent replacement of the pickup coil array without compromising SNR or parallel imaging performance. For different anatomical applications, only the wireless coil array (metasurface) needs to be designed accordingly. Owing to its simple structure—free of the bulky cable assemblies and additional RF components (e.g., baluns, cable traps) required by traditional coils—the wireless coil array can more effectively conform to the region of interest, thereby maximizing SNR performance. Meanwhile, the high‐density pickup coils allow the NFCA to achieve parallel imaging capabilities that are on par with those of conventional coils. The simple structure of the wireless coil arrays also offer a lightweight design and come at a lower cost, which enhances patient comfort and enables customization of the wireless coil array, making them disposable. Customization ensures that the wireless coil arrays can adapt to each individual examination site, maximizing SNR performance, while disposability allows the examination to be performed in a more hygienic environment.

To validate the feasibility of the NFCA concept and address concerns about potential interference between multiple pickup coil elements and multiple wireless coil elements, which could affect performance, as well as to provide design guidance for the wireless coil array, we derived the SNR formula and verified its accuracy through experiments. In vivo studies show that the NFCA case in this study achieves an average SNR improvement of 66% over a traditional knee coil. Moreover, compared to the knee coil, the NFCA achieves the same parallel imaging capability as the conventional coil at 2× acceleration.

As wireless coils progress toward clinical application, achieving high SNR and robust parallel imaging capability are paramount. High SNR is essential for acquiring high‐contrast, high‐resolution MRI images, and it also offsets the SNR degradation associated with parallel imaging techniques. Parallel imaging capability, conversely, plays a pivotal role in reducing scan durations. This reduction is crucial for minimizing SAR deposition, improving patient comfort, and easing the demand on MRI resources.

In terms of SNR, one of the primary contributions of this work is the derivation and validation of the SNR expression for an *m* × *n* element NFCA. This expression reveals two key insights: 1) The signal distribution in the NFCA is determined by the weighted combination of the B1 fields from each wireless coil element (metamaterial), with the weighting coefficients based on the mutual inductance between each element and all pickup coil elements. 2) When the B1 field coefficients of the wireless coil elements are equal, the SNR expression for the NFCA closely resembles that of a wired coil array of the same dimensions. The first key point indicates that when designing metasurfaces to be used as wireless coils, one can refer to the design principles of wired coil elements and adjust the diameter of the metasurface elements to be comparable to the radius of the target area. This ensures sufficient B1 field penetration across the entire knee region. In this study, the metasurface was designed using this approach, with element diameters appropriately matched to the size of the knee. The second point suggests that the NFCA may offer an SNR advantage compared to traditional rigid coils due to the closer proximity of the wireless coil to the patient. This formula is broadly applicable to various decoupling techniques between wireless coil elements (metasurfaces), including not only the wireless coil array described here, but also, in theory, other coil arrays or metasurfaces decoupled by alternative approaches.

In terms of parallel imaging performance, simulation and experimental results suggest that the decoupled metamaterial may introduce additional diversity in the spatial distribution of signals beyond that provided by the original pickup coil array. When combined with the multichannel pickup coil array used in this study, this characteristic may offer the potential to achieve clinically viable parallel imaging performance.

It should be noted, however, that the study described in this work has certain limitations. The pickup coil array used in our experiments is composed of existing surface coils and spinal coils, rather than a specially designed universal coil. This implies that the NFCA in our study did not achieve its optimal performance. Fortunately, this does not affect the validity of our conclusions.

## 5 Conclusion

In this paper, we introduced the coil architecture of the NFCA and derived its theoretical SNR formula. The simulation and experimental results obtained from the phantom study were found to be consistent with theoretical expectations. Experimental results show that the NFCA case in this study achieves an average SNR improvement of 66% compared to the commercial knee coil, with the average acceleration factor at 2× acceleration being equivalent to that of the commercial coil.

In conclusion, the theoretical analysis and case experiments in this paper demonstrate that this theoretical framework has the potential to replace traditional wired coils. This could pave the way for the future shift in clinical magnetic resonance imaging RF coils, from bulky traditional wired coil arrays to lightweight metasurfaces that do not require cable connections. This shift may revolutionize the workflow of clinical MRI scanning, significantly enhancing both efficiency and the examination experience.

## 6 Experimental Section

### Fabrication of the Wireless Coil

**Figure 7:**
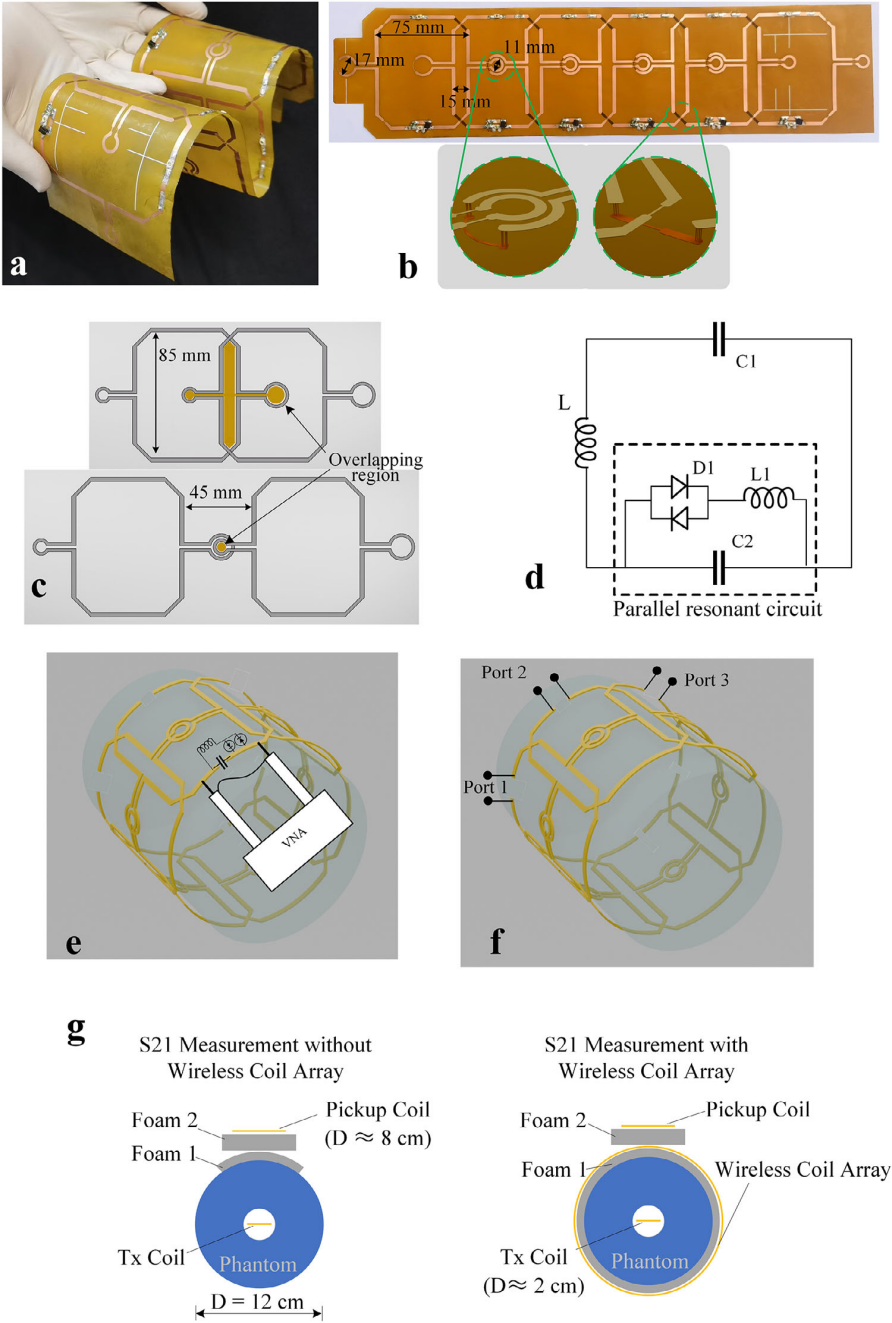
Schematic of the wireless coil array (metasurface). a) Wireless coil array. b) Detailed view of the overlapping area between adjacent coils. c) Schematic illustration of the overlap region. d) Circuit diagram of a wireless coil element. e) Detuning characteristics measured with dual probes (VNA: vector network analyzer). f) Open‐ended ports used for measuring inter‐element coupling. g) Schematic of the experimental setup for evaluating the contribution of the wireless coil array to signal transmission.

The metasurface was fabricated using flexible printed circuit (FPC) boards with a 50 µm polyimide substrate and 35 µm thick copper. Its dimensions are illustrated in **Figure** **7**a. The main trace width is 3 mm, with an overlapping trace width of 0.5 mm between adjacent elements. Each coil element incorporates a variable capacitor (Voltronics, JZ200) with a range of 4.5–20 pF, two diodes (Macom, UM9989), and a 100 µH inductor.

As shown in Figure 7b, the units are connected by vias, avoid electrical connections between adjacent and next‐nearest neighboring coil elements. An overlapping region exists between adjacent and next‐adjacent elements (Figure 7c). This region has been intentionally designed to ensure that the magnetic flux passing through it counteracts the flux from other parts of the coil, thereby minimizing coupling. Figure 7d shows the equivalent circuit diagram of a wireless coil element. During the RF transmission phase, the induced electromotive force turns on the bidirectional diode D1, causing L1 and C2 to resonate in parallel. This places the module in a high‐impedance state, also known as the detuned state, thereby reducing its impact on the transmit field and the SAR.

As demonstrated by the theoretical derivation, the total B1 field of the NFCA is a weighted combination of the B1 fields from individual elements. Drawing upon prior design experience with conventional wired RF coils, it is generally preferable for the diameter of each element to be comparable to the radius of the target anatomy to ensure adequate penetration depth. Accordingly, the lateral dimension of the wireless coil was set to ≈70 mm.

### Bench Testing

For detuning measurements, the coil loops were opened, and a 1 V voltage was applied across the diodes to activate them. The ground terminals of both VNA ports were connected, and the ports were attached to each end of the detuning circuits (Figure 7e). For inter‐element coupling measurements, each unit was set to resonance, the loops were opened, and the two ends were connected to the signal and ground of one VNA port (Figure 7f). Magnetic responses were measured using a dual‐loop decoupled probe. All tests were performed under loaded conditions. To verify the amplification effect of the wireless coil array during signal transmission, the transmission coefficient S21 was measured at different distances with and without the wireless coil array. In the experimental setup shown in Figure 7g, a single‐loop probe with a diameter of ≈2 cm was used as the transmit coil (Tx coil). More detailed information can be found in Text S1 of the Supporting Information.

### 6.1 Electromagnetic Simulations for Signal Conbination Validation

The NFCA used to receive signals consists of the 6‐element wireless coil array (metasurface) combined with a row of 4‐element pickup coil array. First, the mutual inductance between each wireless coil element and the pickup coil element was calculated from the simulation results, and then the B1 maps were computed using a cosimulation approach.^[^ ^45^ ^]^ The sample noise was calculated based on the electric field distribution of the wireless coils within the phantom. The SNR distribution was then obtained by dividing the B1 field by the standard deviation of the sample noise, and subsequently normalized to its maximum value. The simulations were performed using CST Studio Suite 2023. The diameter of the phantom is ≈11 cm. The conductivity and dielectric constant of the phantom were set to 0.52 and 78, respectively.

### Phantom Experiment for SNR Combination Validation

The same setup was used as in the simulations to conduct the phantom experiment, as shown in Figure 4. The four spine coils in the bed were employed as the pickup coils. P2 and P3 exhibit stronger coupling with the wireless coil array than P3 and P4, allowing to validate the relationship between SNR and mutual inductance coefficients in the NFCA.

A phantom (Shanghai United Imaging Healthcare) with a diameter of 11 cm and a length of 45 cm, containing a solution with 1.243 g NiSO_4_·6H_2_O and 2.6 g NaCl per 1000 g of water was used in the experiment. Turbo spin‐echo (TSE) sequences were conducted with the following parameters: field of view (FOV) = 220 mm × 220 mm, slice thickness = 3 mm, voxel size = 0.86 × 0.86 × 3 mm^3^, repetition time (TR) = 5327 ms, echo time (TE) = 78.96 ms, bandwidth = 300 Hz/pixel, flip angle (FA) = 90°, and number of averages (NSA) = 1. All MRI experiments in this study were conducted on a 3T system (uMR 790, Shanghai United Imaging Healthcare, Shanghai, China).

### 3D Electromagnetic Simulations for SAR Analysis

A shielded transmit coil (VTC) from a 3T MRI system was constructed, along with the NFCA. Both the pickup coil array and the wireless coil array were modeled in a detuned state. The wireless coil array was positioned ≈0.5–1 cm away from the knee, which corresponds to the combined thickness of typical clothing and the potential housing of the coil. The simulation power was set to 1 W.

### Phantom Experiment for SAR Analysis

A phantom with a diameter of ≈12 cm and a height of about 25 cm was fabricated for thermal testing and SAR analysis. The phantom solution consisted of sodium chloride (NaCl) and hydroxyethyl cellulose (HEC) at concentrations of 1.55 and 31 g L^−1^, respectively.^[^ ^46^ ^]^ To simulate extreme conditions, a duty cycle sequence (TR = 100 ms, RF duty cycle = 4%, transmit field strength B1^+^ = 22 µT) was applied for 30 min. Twelve infrared fiber optic temperature probes (OmniFlex‐2, Neoptix Canada LP, Canada) were positioned at hotspot locations identified by simulation to measure temperature rise. Local SAR values were calculated based on the measured temperature rise^[^ ^47^ ^]^

(13) $$SAR=\frac{\Delta T\cdot C}{\tau }$$

where Δ*T* is the temperature rise, *C* is the specific heat capacity of the phantom, and *τ* is the duration.

### In Vivo Study

In this in vivo study, 9 adult male volunteers were involved, and the experiment was conducted in accordance with protocols approved by the Human Research Ethics Committee at the Shenzhen Institute of Advanced Technology (SIAT), Chinese Academy of Sciences (CAS), China (Project number: SIAT‐IRB‐240715‐H0897). Informed written consent was obtained from all participants prior to the research. The experiment had the same volunteer lie in a supine position on the bed, with imaging of the knee performed using both the knee coil and the NFCA shown in Figure 5a,b. Compared to the NFCA used in the phantom experiment, this NFCA includes an additional 12‐channel receive‐only surface coil (Knee Coil – 12, United Imaging Healthcare, Shanghai, China) as the pickup coil array to enable circumferential signal acquisition. In the study, spin‐echo sequences were used with the following parameters: field of view (FOV) = 120 mm × 120 mm, slice thickness = 3 mm, voxel size = 0.94 × 0.94 × 3 mm^3^, repetition time (TR) = 6809 ms, echo time (TE) = 94.2 ms, bandwidth = 250 Hz/pixel, flip angle (FA) = 90°, and number of averages (NSA) = 1 to acquire knee cross‐sectional images. The results were used to calculate the SNR and g‐factors using the SENSE algorithm. A gradient echo (GRE) sequence with the same parameters was used to acquire noise data. For high‐resolution imaging, the T2‐weighted spin‐echo sequence was used with the following parameters: FOV = 120 mm × 120 mm, slice thickness = 2 mm, voxel size = 0.33 × 0.33 × 3 mm^3^, TR = 4436 ms, TE = 139.2 ms, bandwidth = 250 Hz/pixel, autocalibration signal (ACS) = 2.22, scan time = 2:49 min, flip angle (FA) = 90°, and NSA = 1.

### Statistical Analysis

The normality of the SNR data for both the NFCA and the knee coil conditions was assessed using the Lilliefors test. The resulting *p*‐values of 0.37 (NFCA) and 0.35 (knee coil) both exceeded the significance threshold of 0.05, indicating no evidence to reject the null hypothesis of normal distribution. This supports the appropriateness of using parametric statistical methods for subsequent analyses.

The SNR improvement with NFCA was statistically significant (*p* = 5.6 × 10^−^⁵ < *α* = 0.05, paired *t*‐test), observed uniformly in the cohort (*n* = 9). The data are presented as mean ± standard deviation (SD): 211 ± 41.4 for NFCA and 127 ± 21.2 for the knee coil. The SNR values used in the analysis were obtained from the middle slice of the three acquired slices for each subject.

All statistical analyses were conducted using MATLAB (MathWorks, Natick, MA). The mean values were rounded to the nearest integer, and standard deviations were rounded to one decimal place using conventional rounding rules. No data transformation or outlier exclusion was applied.

The participant group (*N* = 9) included both male and female volunteers, aged 25–62 years (mean ± SD: 32.6 ± 11.4), with body weights ranging from 46 to 75 kg (mean ± SD: 56.2 ± 9.8) and heights ranging from 156 to 181 cm (mean ± SD: 164.8 ± 9.1).

## Conflict of Interest

The authors declare no conflict of interest.

## Supporting information



Supporting Information

## Data Availability

The data that support the findings of this study are available from the corresponding author upon reasonable request.
