# Supplementary material for: A Near‐Field Coupling Array Enables Parallel Imaging and SNR Gain in MRI
Source: Adv Sci (Weinh). 2025 Jul 22;12(37):e03481. doi: 10.1002/advs.202503481 (PMC12499448; doi:10.1002/advs.202503481)
Supplement: Supplementary file 1 — Supporting Information [file ADVS-12-e03481-s001.docx]

Supporting Information

Text S1. Intuitive Explanation of NFCA’s SNR Advantage Over the Knee Coil


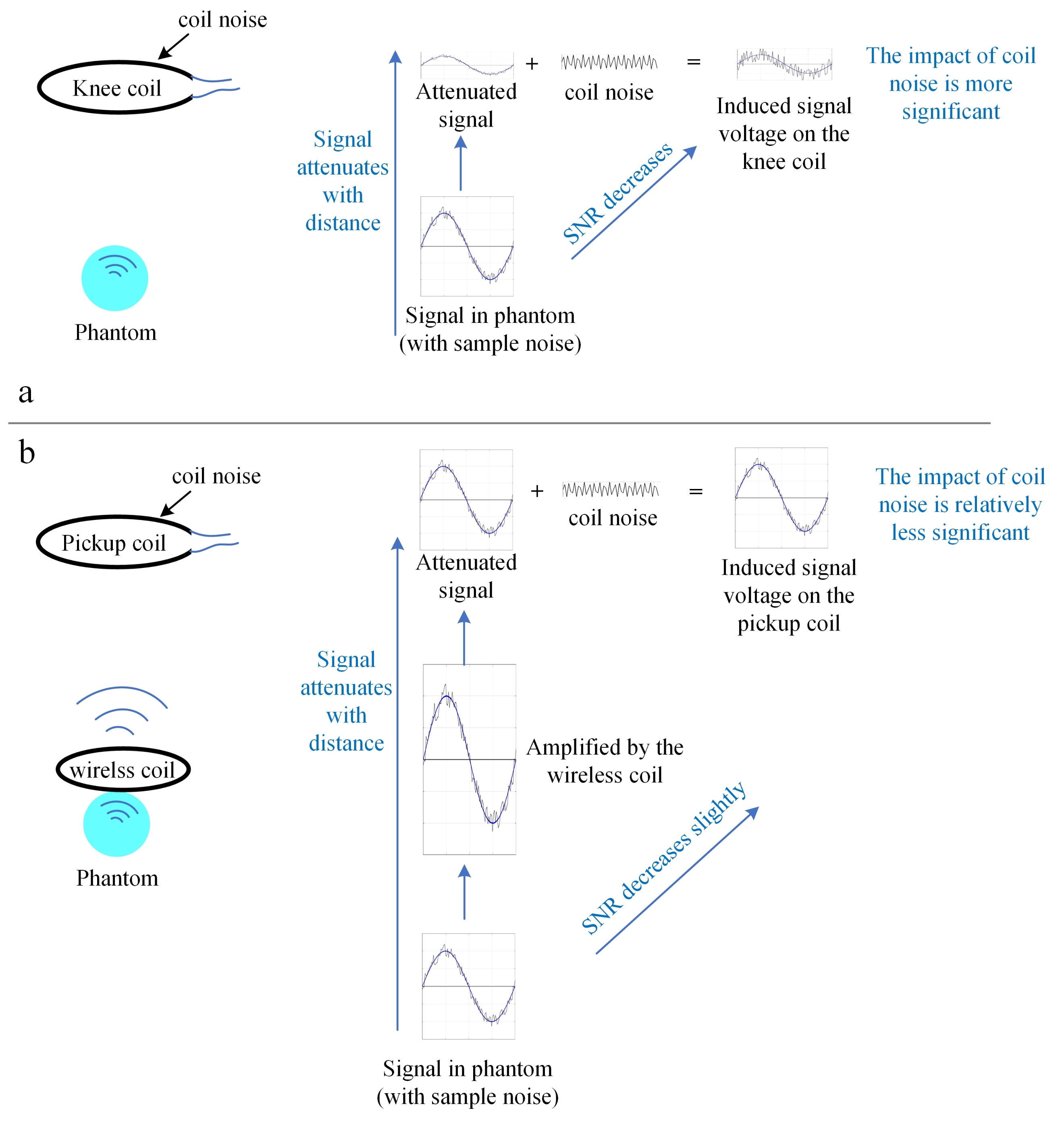


**Figure S1**. Signal propagation diagram. a. Phantom with a poorly conforming knee coil.

b. Phantom and NFCA.

**For traditional wired coils, the SNR behavior is as follows:** As shown in **Figure S1a**, the signal originating from the phantom, which includes sample noise, attenuates rapidly with increasing propagation distance. The greater the distance between the coil and the imaging region, the smaller the amplitude of the electromotive force induced on the coil. When this weakened signal is combined with the coil noise, the resulting signal-to-noise ratio (SNR) is further reduced. In other words, the farther the distance, the more pronounced the impact of coil noise on SNR becomes. This phenomenon fundamentally explains why the SNR of conventional coils decreases as the distance from the imaging target increases.

**For the NFCA, the SNR behaves as follows:** As shown in **Figure S1b**, for the NFCA, although the pickup coils are located farther from the phantom compared to the knee coil, the wireless coils are positioned closer to the phantom. When the signal propagates to the wireless coil, the resonant wireless coil acts as a spatial RF signal amplifier. After amplification, the signal reaching the pickup coil is substantially increased, making it significantly stronger relative to the coil noise. Consequently, the degrading effect of coil noise on the SNR is mitigated.

To verify that the wireless coil array indeed has a signal amplification effect, we conducted the following experiment. The experimental setup is shown in **Figure S2**.


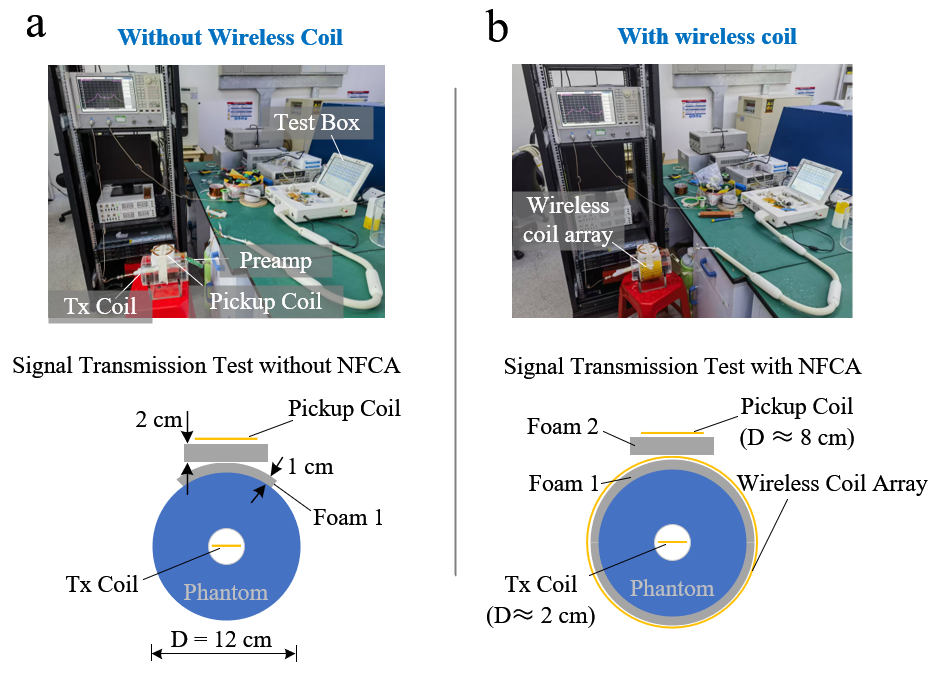


**Figure S2**. Experimental setup for S21 measurement and cross-sectional schematic illustrating the phantom and coil configuration. (a) Without the wireless coil. (b) With the wireless coil. The image shows the case where two pieces of foam are placed between the pickup coil and the phantom (3 cm).

Port 1 of the vector network analyzer (VNA) was connected to a coil used as the transmit coil (Tx), while Port 2 was connected to a custom-built test box. This test box supplies power to the pickup coil, enabling it to operate in receive mode, and transmits the signal received by the pickup coil ( ~8 cm diameter) back to Port 2 of the VNA. A cable connected to the test box was further linked to a preamplifier and a single-channel pickup coil. During the S21 measurement, the signal is transmitted by the Tx coil connected to Port 1, picked up by the pickup coil, amplified by the RF preamplifier, passed through the test box, and then returned to Port 2 of the VNA.

In the experiment, we employed a cylindrical phantom filled with 0.9% saline solution, approximately 12 cm in diameter and 20 cm in length. A through-hole with a diameter of about 3 cm was present at the center of the phantom, allowing insertion of the Tx coil. To adjust the distance between the phantom and the pickup coil, foam layers of varying thicknesses were placed in between to fix the separation. By changing the number of foam layers, we measured the S21 at different distances, and observed the variation in S21 with and without the presence of the wireless coil. Except for Foam 1 with a thickness of approximately 1 cm, all other foam layers were about 2 cm thick.


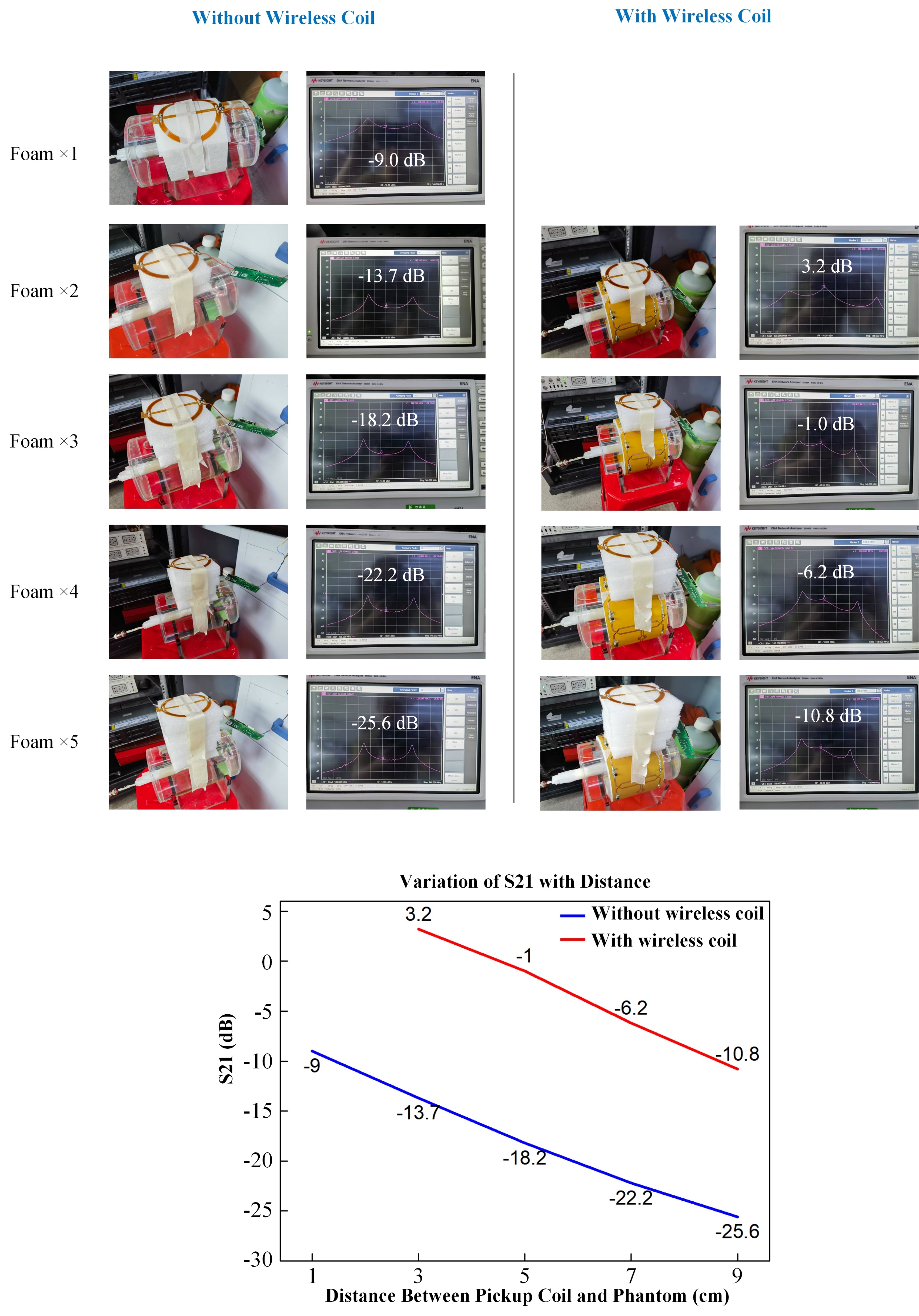


**Figure S3**. S21 versus distance between pickup coil and phantom, with and without wireless coil

The measured results are shown in **Figure S3**. The results indicate that when the distance between the pickup coil and the phantom was 3 cm, 5 cm, 7 cm, and 9 cm, the presence of the wireless coil array led to an improvement of 15–17 dB in the S21 of the pickup coil. **This indicates that the wireless coil array indeed exhibits a significant signal amplification effect.**

Figure S4: Schematic diagram of the setup for coupling ratio measurement.


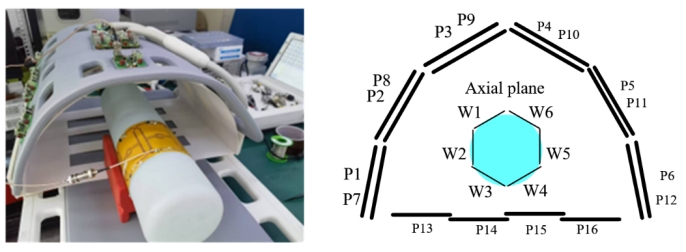


**Table S1**. Coupling ratios (S21, in dB) between the pickup coils and the wireless coils.

|  | W1 | W2 | W3 | W4 | W5 | W6 |
| --- | --- | --- | --- | --- | --- | --- |
| P1 | -30 | -32 | -26 | -30 | -29 | -27 |
| P2 | -21 | -14 | -28 | -26 | -28 | -24 |
| P3 | -9 | -15 | -24 | -23 | -28 | -18 |
| P4 | -16 | -25 | -22 | -25 | -19 | -8 |
| P5 | -28 | -29 | -26 | -29 | -13 | -20 |
| P6 | -36 | -35 | -29 | -25 | -31 | -34 |
| P7 | -31 | -31 | -26 | -31 | -28 | -28 |
| P8 | -20 | -15 | -29 | -28 | -24 | -22 |
| P9 | -9 | -14 | -24 | -22 | -19 | -15 |
| P10 | -14 | -24 | -23 | -23 | -16 | -10 |
| P11 | -29 | -27 | -28 | -32 | -12 | -21 |
| P12 | -38 | -33 | -32 | -27 | -29 | -29 |
| P13 | -30 | -23 | -21 | -29 | -33 | -33 |
| P14 | -30 | -20 | -9 | -25 | -31 | -34 |
| P15 | -36 | -33 | -26 | -8 | -21 | -28 |
| P16 | -33 | -36 | -29 | -23 | -24 | -29 |

Note: P1–P16 denote the pickup coils, and W1–W6 denote the wireless coils.

**Figure S5: Temperature rises measured by the 12 fiber-optic probes, with the maximum increase being approximately 1.1 °C.**


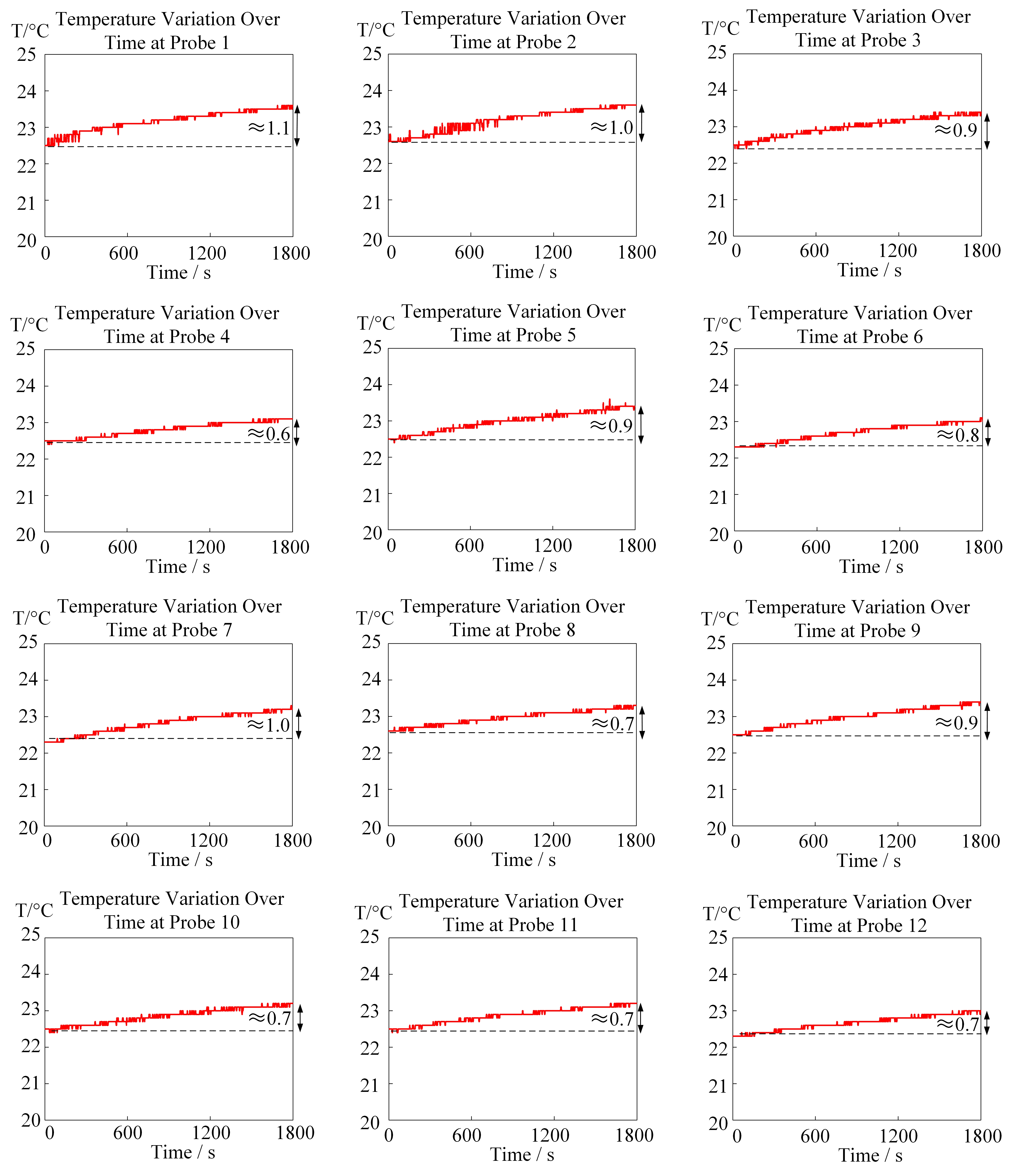


**Table S2**. The meanings of symbols in the text.

| Symbol | Meaning |
| --- | --- |
| 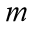 | The total number of elements in the pickup coil array. |
| 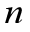 | The total number of elements in the wireless coil array. |
| *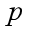* | p is a variable representing a wireless coil, ranges from 1 to n. |
| *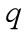* | p is a variable representing a pickup coil, ranges from 1 to m. |
| 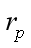 | The coil resistance of the p-th wireless coil. |
| 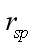 | The equivalent sample noise resistance of the p-th wireless coil. |
| 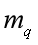 | It is a subscript representing the q-th pickup coil. |
| 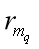 | The coil resistance of the q-th pickup coil. |
| 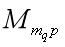 | The mutual inductance coefficient between the p-th wireless coil and the q-th pickup coil. |
| 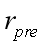 | The input impedance of the preamplifier. |
| 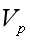 | The signal voltage induced on the p-th wireless coil. |
| 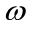 | The operating angular frequency of the system. |
| 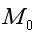 | Magnetization density. |
| 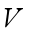 | The volume of the voxel. |
| 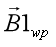 | The transverse magnetic field generated by unit current in the p-th wireless coil. |
| 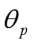 | The angle of the RF magnetic field measured from some fixed reference in the laboratory frame. |
| 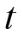 | Time in seconds. |
| 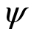 | The arbitrary phase of the rotating nuclei. |
| 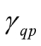 | A scaling factor that quantifies the change in amplitude of signal voltage. |
| 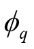 | The phase change experienced by the q-th pickup coil as it transmits to the system. |
| 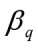 | The amplification of the signal voltage from the q-th pickup coil to the system. |
| 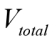 | The sum of the signal voltages obtained by all pickup coils. |
| 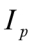 | The induced current on the p-th wireless coil. |
| 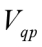 | The signal voltage on the q-th pickup coil caused by the p-th wireless coil. |
| 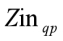 | The input impedance seen by the q-th pickup coil due to the p-th wireless coil. |
| 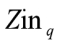 | The total imput impedance of the q-th pickup coil. |
| 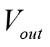 | The amplitude of the coil's output voltage. |
| 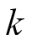 | Boltzmann constant. |
| 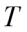 | Kelvin temperature. |
| 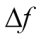 | The system's receiver bandwidth. |
| 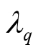 | A coefficient used to reflect the influence of the q-th pickup coil on the input impedance. |
| 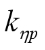 | The coefficient of the B1 field of the p-th wireless coil in the case of a single pickup coil. |
| 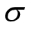 | Conductivity. |
| 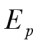 | The spatially dependent electric field distribution generated by the p-th wireless coil. |
| 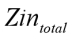 | The total input impedance seen from all pickup coils. |
| 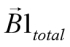 | The total B1 field seen from all pickup coils. |
| 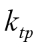 | The coefficient of the B1 field of the p-th wireless coil in the case of the pickup coil array. |
| 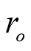 | The resistance on the equivalent loop of sample noise. |
| 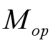 | The mutual inductance coefficient between the sample noise and the p-th wireless coil. |
| 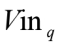 | The input voltage of the q-th pickup coil. |
| 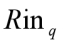 | The input impedence of the q-th pickup coil. |
| 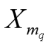 | The imaginary part of the impedance on the q-th pickup coil. |
| 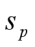 | The coil conductance of the p-th wireless coil. |
| 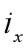 | The current of the ideal current loop. |
| 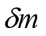 | The magnetic moment of the ideal current loop. |
| 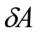 | The area of the ideal current loop. |
| 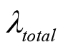 | A coefficient used to reflect the influence of the pickup coil array on the input impedance. |
